# A Novel Position and Orientation Sensor for Indoor Navigation Based on Linear CCDs

**DOI:** 10.3390/s20030748

**Published:** 2020-01-29

**Authors:** Chuang Wang, Li Xing, Xiaowei Tu

**Affiliations:** 1 School of Mechatronics Engineering and Automation, Shanghai University, Shanghai 200444, China; wangchuang61@shu.edu.cn (C.W.); xinglishu@shu.edu.cn (L.X.); 2 School of Information Engineering, Xuchang University, Xuchang 461000, China

**Keywords:** indoor navigation, orientation, Rodrigues coordinate transformation, cylindrical lens, linear CCD

## Abstract

The position and orientation of a mobile agent, such as robot or drone, etc., should be estimated in a timely way during operation in the structured indoor environment, so as to ensure the security and efficiency of task execution. Concerning the problem that the position and orientation are often estimated separately by different kinds of sensors in the off-the-shelf methods, we design a novel position orientation sensor (POS). The POS consists of four pairs of linear charge-coupled devices (CCDs) and cylindrical lenses, which can estimate the 3D coordinate of the anchor in the POS’s field of view. After detecting at least three anchors in its field of vision sequentially, the Rodrigues coordinate transformation algorithm is utilized to estimate the position and orientation of POS simultaneously. Meanwhile, the position and orientation are estimated at the receiver side. Hence there is no privacy concern associated with this system. The architecture of the proposed POS is symmetrical and redundant, even if one of the linear CCDs or cylindrical lens malfunctions, the whole system could still work normally. The proposed method is cost-effective and easily extends to a wide range. The numerical simulation demonstrates the feasibility and high accuracy of the proposed method, and it outperforms the off-the-shelf methods.

## 1. Introduction

Indoor mobile agents are widely used in various industries, such as logistics environments [1], military applications [2], automated manufacturing [3], commerce [4], etc. One of the most crucial functions of the indoor mobile agent is to accomplish user-specified tasks. Therefore, highly accurate position and orientation information about the mobile agent is a basis for subsequent precise motion control and path planning in the navigation process.

In the past decades, many methods have been proposed for indoor navigation, which can be roughly divided into two categories: relative positioning and absolute positioning [5]. Relative positioning methods estimate the current state based on measuring the distance and orientation of the robot relative to the initial state. Dead reckoning is a classical relative positioning technology method. It uses an inertial measurement unit (IMU) or odometer to realize navigation, and it does not need to transmit or receive any external information. The dead reckoning method can work independently and continuously to provide positioning services for mobile agents. However, the drift errors and noises of the IMU significantly affect the accuracy of the navigation over a large period of time [6], which makes this method only meet the state estimation requirements for a short time, and it cannot realize long-time navigation tasks in a complex environment. Although many improvements have been made to reduce the drift errors and noises, it cannot be applied alone for a long period without any correction strategy. Meanwhile, the dead reckoning method needs to know the initial position and orientation, which is difficult to achieve in actual scenes. Therefore, the estimation of positioning and orientation tends to depend more on absolute positioning methods. 

Absolute positioning has attracted much attention for a long time and has made significant progress. It requires multiple reference points to determine the location of the moving agent. A typical representative of outdoor absolute positioning technology is the global navigation satellite system (GNSS). The outdoor positioning technology has been developed rapidly since the GNSS was put into service. We can observe its convenience when driving a car or using location sharing employing the GNSS module embedded in a smartphone. However, compared with the satellite channel of the GNSS outdoors, indoor positioning faces the terrestrial channel problems, which make it more complicated. In urban areas or inside buildings, walls and roofs can block the electronic waves from satellites, which makes GNSS only a practical solution for positioning in the indoor environment. Since GNSS does not work normally inside buildings, various studies have focused on indoor absolute positioning, and indoor absolute positioning has entered a new period of great changes and developments [7].

Indoor absolute positioning is a hot topic. It has been intensively researched for decades due to its importance and difficulty [8]. During the past few decades, many methods have been proposed to realize indoor positioning, such as Wi-Fi [9], Ultra-Wide Band (UWB) [10], ultrasound-assisted [11], Bluetooth [12], etc. Moreover, some of these methods such as the UWB can achieve cm-level positioning accuracy. However, these positioning techniques often suffer interference from Non-Line-of-Sight signal propagation and multipath fading effects. Meanwhile, they cannot provide three-dimensional (3D) position and orientation simultaneously, which are significant defects in the modern navigation applications of indoor mobile agents.

The photoelectric measurement technology has developed rapidly and has been used in a variety of indoor environments, and many feasible methods have been proposed. Laser radar mainly uses an infrared laser beam to scan the surrounding environment with a radial field of view. It is widely used in indoor mobile agent navigation because of its flexibility and real-time measurement capability. However, in some cases, such as in the assembly and docking procedures of automobile and large aircraft components, it is difficult for the laser radar to estimate the position and orientation [13,14]. In [15], a laser-based photoelectric scanning method is used to measure the 3D coordinate of anchors, and then estimate the position and orientation by using a coordinate transformation algorithm. Nevertheless, this method needs to use a sophisticated rotating laser scanning instrument, which is costly and not convenient for large-scale use in the case of multiple indoor mobile agents. 

The vision sensor based on charge-coupled device (CCD) or CMOS (Complementary Metal Oxide Semiconductor) adopts a non-contact measurement method, which can estimate the motion parameters of indoor moving objects without disturbing the system. In [16], a method using the area CCD is presented to estimate the position and orientation. However, in such a system, the 2D and 3D situations should be considered, respectively, and then the number of different anchors will be decided. In [17], a robot equipped with a camera can be controlled to observe a rectangle object constraint, and then obtain the position and pose. Nevertheless, this system requires that the optical axis of the camera always point to the target center point, which makes the method complex and clumsy. In [18], a CMOS camera based on Wiimote is used to capture infrared LEDs (Light Emitting Diodes) for indoor mobile robot tracking, then the position and orientation of Wiimote can be estimated by using coordinate transformation, but this method is only suitable for 2D scenarios. The ToF camera is also can be used for indoor positioning [19,20], but it cannot work alone for indoor positioning, and needs the assistance of other equipment. Meanwhile, it also cannot estimate the position and orientation simultaneously. 

Currently, there are some off-the-shelf commercial devices to measure the position and orientation information of the indoor mobile target using computer vision, such as the VICON [21]. These systems seem to be the right solution for the navigation of the indoor mobile agent, but such devices are not suitable for changing indoor backgrounds as they fail to work in dark or untextured areas. Meanwhile, these systems are expensive and cannot be easily extended on a large scale. A broader measure space means more requirement of camera measurement equipment, which leads to a higher cost overhead.

The existing visual measurement methods of indoor position and orientation mainly use area CCD or CMOS, and seldom use linear CCD to realize the position and orientation measurement of indoor mobile agents. In [22], the linear CCD is used to measure the position and orientation of moving objects, but this method needs to use multiple sets of linear CCD measuring equipment. Meanwhile, the position and attitude can only be estimated in a small range of indoor spaces. [23] proposed a 3D motion tracking system by using multiple linear optical sensor arrays, supplemented by an IMU, to achieves better performance in position and orientation measurement. However, this method requires the usage of the linear CCD and the IMU to estimate position and attitude, respectively. Meanwhile, it can also only achieve position and orientation measurements in a small range of indoor spaces. 

Currently, there is still a lack of a cost-effective, economical, and satisfactory solution to realize indoor positioning and attitude estimation concurrently. The key to achieving high accuracy indoor position and orientation estimation in an affordable and wide range system is to use the appropriate sensor(s). Motivated by the specific requirement of the indoor position and orientation problem in the navigation application, we design a novel position orientation sensor (POS). This POS can create a 3D coordinate measurement system based on the intersection of four planes by using four pairs of linear CCDs and the cylindrical lenses. We name this created 3D coordinate system of the POS as the position orientation sensor coordinate system (POSCS). Compared with the traditional indoor sensor, which can estimate the distance or angle between the anchors and the mobile target, our method can estimate the 3D coordinate of the anchor in the field of vision (FOV) of the POS. Then according to at least three anchors in the indoor coordinate system (ICS) and their estimated coordinate value in the POSCS, we can use the Rodrigues coordinate transformation algorithm to calculate the rotation vector and translation vector of the POS. The translation vector contains the position of POS in the ICS, and the rotation vector contains the orientation of POS in the ICS. Finally, we can estimate the position and orientation of the indoor mobile agent simultaneously. 

The rest of the paper is organized as follows: In Section 2, the system components and the working principle of POS are introduced, and then the FOV of the designed POS is simulated. In Section 3, the Rodrigues coordinate transformation algorithm is presented to estimate position and orientation. In Section 4, the simulation is performed to demonstrate the feasibility and high accuracy of the proposed method. In Section 5, a brief overview and advantages of the proposed method are discussed. Finally, the main conclusions of the whole work and future improvements are summarized in Section 6.

## 2. Principle of Measurement

### 2.1. System Component and Working Principle

The linear sensor which we use consists of two major components, a linear CCD and a cylindrical lens. The cylindrical lens is used to form the projection mapping from 3D space to the 1D image. According to the basic principles of optics, the rays from an object point on one side of the cylindrical lens forms a linear image on the other side [24].

**Figure 1:**
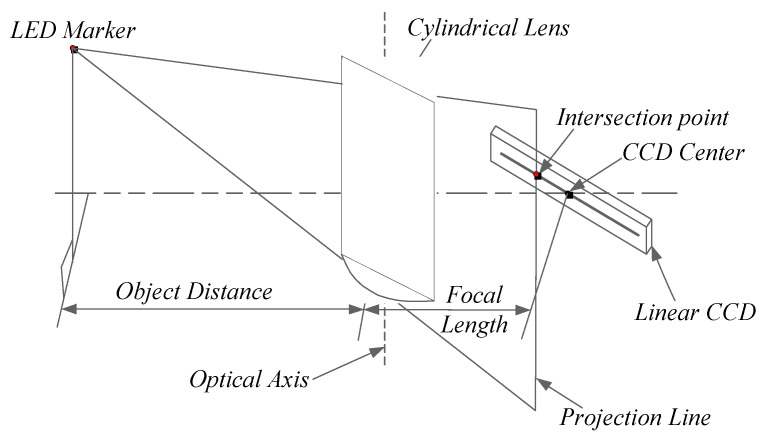
Projection mapping model of the one-dimensional imaging unit.

As shown in Figure 1, a linear CCD and a cylindrical lens constitute a one-dimensional imaging unit (ODIU); the light from the light emitted diode (LED) marker passing through the cylindrical lens will focus to a line which is coplanar to the marker and optical axis of the lens [25]. To detect the change of plane due to the movement of an LED marker, a linear CCD is used, and it is located at the focal plane of the cylindrical lens. This linear CCD is placed perpendicular to the optic axis of the cylindrical lens to detect the change of the projection line when the LED marker moves. The center of the linear CCD is aligned with the center of the optic axis, and their distance is the focal length of the cylindrical lens. 

The LED marker is projected to form a narrow spot on the linear CCD, and the center of the spot is taken as the image position of the marker. If the spot position on the linear CCD and the optical axis of the cylindrical lens are known, then the supporting plane with them can be calculated, and the LED marker is situated on the same plane. 

As we know, a plane can be defined according to a line and a point that is not on that line. In order to determine the 3-D coordinates of a LED marker in the coordinate system, we need at least three ODIUs to register the LED marker’s 1D image position coordinates, and then reconstruct the 3D spatial coordinates of the anchor [26]. However, with the system composed of only three ODIUs, although it can provide the 3D coordinate information reconstruction of one marker, nevertheless, this system is vulnerable if any ODIU breaks. To make full use of the layout of the existing symmetrical structure on the mobile agent, such as a quadrotor drone who has four arms, we will use four ODIUs to determine the 3D coordinates of an anchor. Meanwhile, it is a redundant system by using four ODIUs, even if an ODIU malfunction, the whole system can still work normally. 

**Figure 2:**
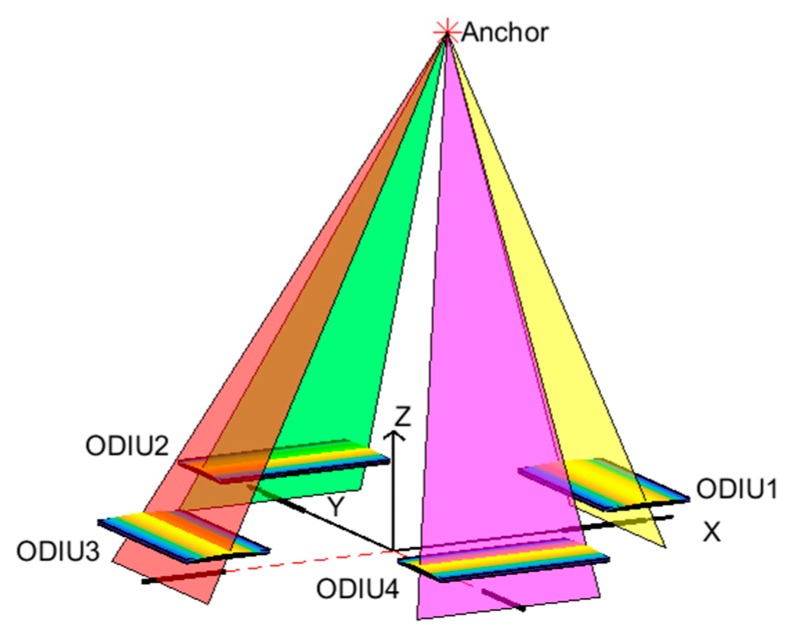
Schematic of the POS.

As shown in Figure 2, four ODIUs are arranged, and the 3D coordinates of the anchor can be reconstructed by the intersection of four planes. When one LED marker (also the anchor) is located in the FOV of the POS, the anchor forms four projection lines crossing the four linear CCDs by passing through the respective cylindrical lens, and the projection lines intersect with the four linear CCD on the focal plane in the respective ODIU. If the position of the projection line in every ODIU is determined, then the plane which contains the anchor is also determined. Thus, the 3D coordinate of the anchor in POSCS can be uniquely resolved by these planes, which is determined by these ODIUs.

Meanwhile, we set the intersection of the four linear CCD’s extension lines as the origin of the POSCS; the photoelectric detection area of every linear CCD is on the XY plane of POSCS. The linear CCD1 and CCD2 are on the positive half-axis of the X-axis and Y-axis, while the CCD3 and CCD4 are on the negative half-axis of the X-axis and Y-axis. According to the created POSCS based on the four ODIUs, once the anchor is located in the FOV of POS, we can estimate the 3D coordinate of the anchor in POSCS. 

### 2.2. Field of Vision of the POS

According to the above introduction of POS, it can only measure the anchor’s coordinate when it is located inside the FOV of the POS. The FOV is very important during the layout design of anchors. Therefore, it is necessary to carry out the FOV simulation for the designed POS. 

**Figure 3:**
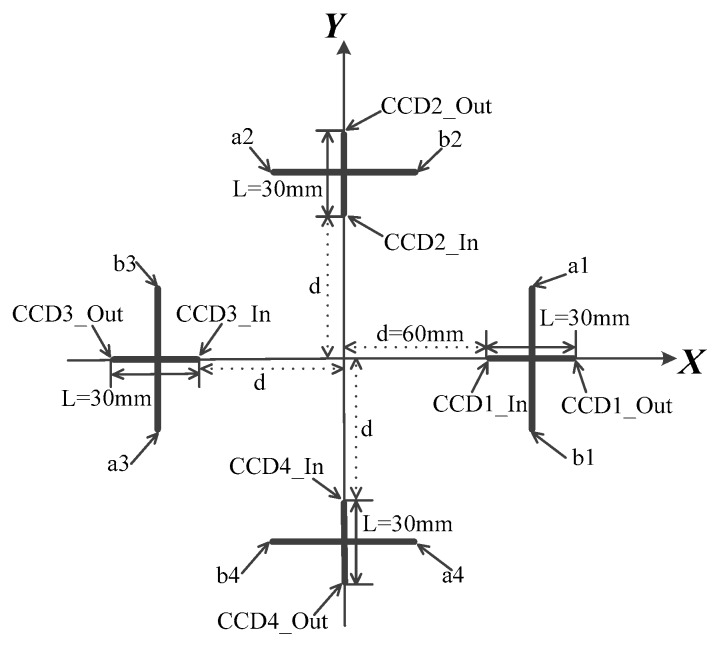
Layout design of the POS.

The structure of the designed POS on the XY plane in POSCS is shown in Figure 3. The sensing length of the linear CCD’s photo-element is 30 mm. The linear CCD1 and CCD3 are on the positive half-axis and negative half-axis of the X-axis, and they are symmetrically placed relative to the origin point, while the CCD2 and CCD4 are on the positive half-axis and negative half-axis of the Y-axis, and they are also symmetrically placed relative to the origin point. 

The CCD1_In, CCD2_In, CCD3_In, and CCD4_In are inner margins of every linear CCD, and their distances d to the origin are 60 mm; the CCD1_Out, CCD2_Out, CCD3_Out, and CCD4_Out are outer margins of the linear CCD, and their distances to the origin are 90 mm. The a1 and b1, a2 and b2, a3 and b3, a4 and b4 are two extreme points on the optical axis of cylindrical lens 1, cylindrical lens 2, cylindrical lens 3, and cylindrical lens 4. The focal length of the cylindrical lens is 50 mm. The values of the above parameters are summarized in Table 1.

**Figure 4:**
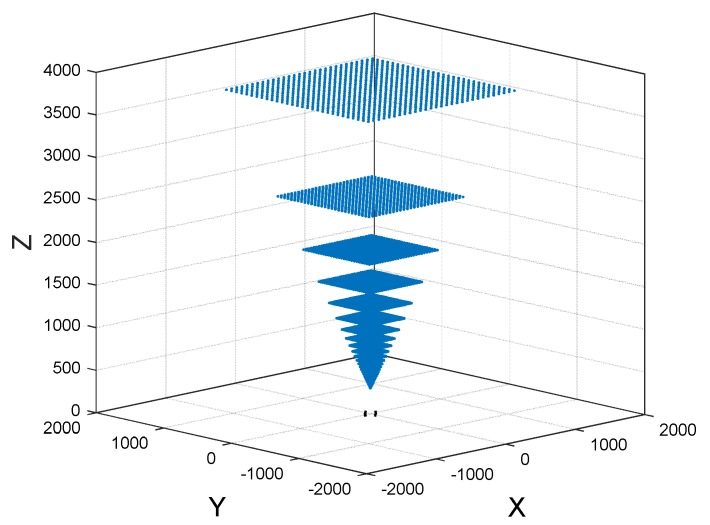
The FOV of the POS.

According to Table 1, we divide the photosensitive area of the four linear CCD every 1 mm to choose the projection line, and there will be many spatial intersections. Here we consider the case that the calculated z value of the spatial intersection below 4000 mm. Figure 4 is the simulated FOV of POS according to the parameters in Table 1. 

In Figure 4, the measurable area of every layer is a square structure, and the closest measurement distance of the POS is 300 mm, that means the distance from an anchor to POS needs to be at least 300 mm if the POS can detect the anchor, meanwhile, the higher of the height, and the broader measurement scope of the POS. 

## 3. Position and Orientation Measurement Algorithm

In our indoor positioning and orientation measurement system, there are two different coordinate systems. One of them is the ICS, and its origin of the coordinate system is set at the corner of the room; while the other is the POSCS and its origin of the coordinate system is set at the POS, as shown in Figure 2. For every POS, it can create a POSCS on its body and then estimates the 3D coordinate of the anchor in its FOV. 

### 3.1. Mathematical Model

Suppose three anchors A_1_, A_2_, A_3_ are fixed on the ceiling, and their coordinates in ICS are A_1_(X_1_, Y_1_, Z_1_), A_2_(X_2_, Y_2_, Z_2_) and A_3_(X_3_, Y_3_, Z_3_). After acquiring the coordinates of three anchors by using POS, and their coordinates in POSCS are C_1_(x_c1_, y_c1_, z_c1_), C_2_(x_c2_, y_c2_, z_c2_) and C_3_(x_c3_, y_c3_, z_c3_). According to the 3D coordinate value of three anchors in POSCS and ICS, three coordinate transformation equations are depicted to realize the coordinate transformation from POSCS to ICS, as shown in Equations (1)–(3):

(1) $$\left[\begin{array}{c} X_{1}\\ Y_{1}\\ Z_{1} \end{array}\right]=R\cdot \left[\begin{array}{c} x_{c1}\\ y_{c1}\\ z_{c1} \end{array}\right]+\left[\begin{array}{c} x_{0}\\ y_{0}\\ z_{0} \end{array}\right],$$

(2) $$\left[\begin{array}{c} X_{2}\\ Y_{2}\\ Z_{2} \end{array}\right]=R\cdot \left[\begin{array}{c} x_{c2}\\ y_{c2}\\ z_{c2} \end{array}\right]+\left[\begin{array}{c} x_{0}\\ y_{0}\\ z_{0} \end{array}\right],$$

(3) $$\left[\begin{array}{c} X_{3}\\ Y_{3}\\ Z_{3} \end{array}\right]=R\cdot \left[\begin{array}{c} x_{c3}\\ y_{c3}\\ z_{c3} \end{array}\right]+\left[\begin{array}{c} x_{0}\\ y_{0}\\ z_{0} \end{array}\right],$$

In (1), (2), (3), we have established the relationship between two coordinate systems for three anchors, where the R is the 3*3 rotation vector, T= [x_0_, y_0_, z_0_]^T^ is the translation vector. In the coordinate transformation equation, the translation vector T contains the origin position of POSCS in ICS, and rotation vector R contains the rotation angle of the POSCS in ICS, which are exactly what we desired. 

Suppose the x, y, and z-axis rotation angle of the POS in ICS are α, β, and γ, respectively. Meanwhile, we set the α as the pitch angle, the β as the roll angle, the γ as the yaw angle, and we define the clockwise rotation angle to be positive and the counterclockwise rotation angle to be negative. Then according to the rotation angle, we can get the rotation matrix that surrounds the x, y, z-axis, as shown in Equations (4), (5), and (6):

(4) $$R_{x}(\alpha )=\left[\begin{array}{ccc} 1 & 0 & 0\\ 0 & \cos\alpha & \sin\alpha \\ 0 & -\sin\alpha & \cos\alpha \end{array}\right],$$

(5) $$R_{y}(\beta )=\left[\begin{array}{ccc} \cos\beta & 0 & -\sin\beta \\ 0 & 1 & 0\\ \sin\beta & 0 & \cos\beta \end{array}\right],$$

(6) $$R_{z}(\gamma )=\left[\begin{array}{ccc} \cos\gamma & \sin\gamma & 0\\ -\sin\gamma & \cos\gamma & 0\\ 0 & 0 & 1 \end{array}\right],$$

We define the rotation vector R = R_y_(β)* R_x_(α)* R_z_(γ), and the result of R is shown in (7), where α = arcsin (R[2, 3]), β = −arctan (R[1, 3]/R[3, 3]), γ = −arctan (R[2, 1]/R[2, 2]):

(7) $$R=\left[\begin{array}{ccc} \cos\beta \cos\gamma -\sin\alpha \sin\beta \sin\gamma & \cos\beta \sin\gamma +\sin\alpha \sin\beta \cos\gamma & -\cos\alpha \sin\beta \\ -\cos\alpha \sin\gamma & \cos\alpha \cos\gamma & \sin\alpha \\ \sin\beta \cos\gamma +\cos\beta \sin\alpha \sin\gamma & \sin\beta \sin\gamma -\cos\beta \sin\alpha \cos\gamma & \cos\alpha \cos\beta \end{array}\right],$$

The key to solving the coordinate transformation in (1), (2), and (3) is to determine the rotation vector R and the translation vector T. If we can acquire the R and T by solving the (1), (2), and (3), that means we can estimate the position and orientation of POS in ICS. So how to solve the rotation vector R and the translation vector T are the key point. 

### 3.2. Rodrigues Coordinate Transformation Algorithm

In order to solve the R and T, three common points are required at least [27]. The 3D coordinate transformation is one of the most frequently encountered operations in geodesy, mapping, photogrammetry, computer vision, geographical informational science, etc. [28]. In this paper, we will elaborate on the Rodrigues coordinate transformation algorithm to solve the R and T.

In (8), R is the rotation vector, T is the translation vector. The parameters to be estimated are R and T. According to the mathematical transformation model, R is computed first, followed by the T:

(8) $$\left[\begin{array}{c} X\\ Y\\ Z \end{array}\right]=R\cdot \left[\begin{array}{c} x\\ y\\ z \end{array}\right]+T,$$

Let the anti-symmetric matrix S is equal to (9), in which a, b, c are independent parameters.

(9) $$S=\left[\begin{array}{ccc} 0 & -c & -b\\ c & 0 & -a\\ b & a & 0 \end{array}\right],$$

The R is composed of anti-symmetric matrix S and 3*3 unit matrix I, as shown in (10).

(10) $$R={\left(I-S\right)}^{-1}*(I+S),$$

From (8), each anchor can list three equations, the equation of the second common point subtract the corresponding equation of the first common point, which can eliminate the translation vector T, then get the Equation (11).

(11) $$\left[\begin{array}{c} X_{2}-X_{1}\\ Y_{2}-Y_{1}\\ Z_{2}-Z_{1} \end{array}\right]=R\cdot \left[\begin{array}{c} x_{2}-x_{1}\\ y_{2}-y_{1}\\ z_{2}-z_{1} \end{array}\right],$$

The Equation (10) into (11), then get (12).

(12) $$(I-S)\cdot \left[\begin{array}{c} X_{2}-X_{1}\\ Y_{2}-Y_{1}\\ Z_{2}-Z_{1} \end{array}\right]=(I+S)\cdot \left[\begin{array}{c} x_{2}-x_{1}\\ y_{2}-y_{1}\\ z_{2}-z_{1} \end{array}\right],$$

We substitute (9) into (12), unfold the equation, then extract the a, b, c expression vector form as shown in formula (13), in which X_12_ = X_2_ − X_1_, Y_12_ = Y_2_ − Y_1_, Z_12_ = Z_2_ − Z_1_; x_12_ = x_2_ − x_1_, y_12_ = y_2_ − y_1_, z_12_ = z_2_ − z_1_:

(13) $$\left[\begin{array}{ccc} 0 & -z_{12}-Z_{12} & -y_{12}-Y_{12}\\ -z_{12}-Z_{12} & 0 & x_{12}+X_{12}\\ y_{12}+Y_{12} & x_{12}+X_{12} & 0 \end{array}\right]\cdot \left[\begin{array}{c} a\\ b\\ c \end{array}\right]=\left[\begin{array}{c} X_{12}-x_{12}\\ Y_{12}-y_{12}\\ Z_{12}-z_{12} \end{array}\right],$$

Obviously, in (13), the left coefficient matrix is a singular matrix and only having two independent equations, which cannot solve a, b, c parameters with two anchors. With the first and the third common points, we can get a similar equation, as shown in (14).

(14) $$\left[\begin{array}{ccc} 0 & -z_{13}-Z_{13} & -y_{13}-Y_{13}\\ -z_{13}-Z_{13} & 0 & x_{13}+X_{13}\\ y_{13}+Y_{13} & x_{13}+X_{13} & 0 \end{array}\right]\cdot \left[\begin{array}{c} a\\ b\\ c \end{array}\right]=\left[\begin{array}{c} X_{13}-x_{13}\\ Y_{13}-y_{13}\\ Z_{13}-z_{13} \end{array}\right],$$

Then we combine (13) and (14), and thus get (15):

(15) $$\left[\begin{array}{ccc} 0 & -z_{12}-Z_{12} & -y_{12}-Y_{12}\\ -z_{12}-Z_{12} & 0 & x_{12}+X_{12}\\ y_{12}+Y_{12} & x_{12}+X_{12} & x_{12}+X_{12}\\ 0 & -z_{13}-Z_{13} & -y_{13}-Y_{13}\\ -z_{13}-Z_{13} & 0 & x_{13}+X_{13}\\ y_{13}+Y_{13} & x_{13}+X_{13} & 0 \end{array}\right]\cdot \left[\begin{array}{c} a\\ b\\ c \end{array}\right]=\left[\begin{array}{c} X_{12}-x_{12}\\ Y_{12}-y_{12}\\ Z_{12}-z_{12}\\ X_{13}-x_{13}\\ Y_{13}-y_{13}\\ Z_{13}-z_{13} \end{array}\right],$$

Solving Equation (15) we can calculate the three parameters a, b, c. According to the value of a, b, and c, we can calculate the value of the rotation vector R by using (10). Then by substituting R into (8), we can calculate the value of the translation vector T. After the above procedure, we can get the R and T, which contain the orientation angle and position of POS in ICS. 

### 3.3. Multiple Common Points in Rodrigues Coordinate Transformation Algorithm

If the POS detects more than three common points within its field of view at a given position, the problem of estimating the rotation vector and the translation vector can be converted to the least square problem, and the error Equation (16) is listed according to Equation (15).

(16) $$V_{3(n-1)\times 1}=A_{3(n-1)\times 3}\cdot X_{3\times 1}-L_{3(n-1)\times 1},$$

In error Equation (16), X_3×1_= [a, b, c]^T^, the matrix of A_3(n−1)×3_ and L_3(n−1)×1_ are shown in Equation (17) and Equation (18).

(17) $$A_{3(n-1)\times 3}=\left[\begin{array}{ccc} 0 & -z_{12}-Z_{12} & -y_{12}-Y_{12}\\ -z_{12}-Z_{12} & 0 & x_{12}+X_{12}\\ y_{12}+Y_{12} & x_{12}+X_{12} & 0\\ \vdots & \vdots & \vdots \\ 0 & -z_{1n}-Z_{1n} & -y_{1n}-Y_{1n}\\ -z_{1n}-Z_{1n} & 0 & x_{1n}+X_{1n}\\ y_{1n}+Y_{1n} & x_{1n}+X_{1n} & 0 \end{array}\right],$$

(18) $$L_{3(n-1)\times 1}={\left[\begin{array}{ccccccc} X_{12}-x_{12} & Y_{12}-y_{12} & Z_{12}-z_{12} & \cdots & X_{1n}-x_{1n} & Y_{1n}-y_{1n} & Z_{1n}-z_{1n} \end{array}\right]}^{T},$$

According to the principle of least squares, the optimal solution of Equation (16) can be obtained, as shown in Equation (19):

(19) $$X={\left(A^{T}\cdot A\right)}^{-1}\cdot A^{T}\cdot L,$$

According to Equation (19), three independent parameters in the Rodrigues coordinate conversion algorithm can be obtained in the case of multiple common points. Based on a, b, and c, the rotation vector R can be obtained by Equations (9) and (10). 

When the measurement error of the common point is considered, the error Equation (20) is obtained according to Equation (8):

(20) $$E=\left[\begin{array}{c} X\\ Y\\ Z \end{array}\right]-R\cdot \left[\begin{array}{c} x\\ y\\ z \end{array}\right]-\left[\begin{array}{c} x_{0}\\ y_{0}\\ z_{0} \end{array}\right],$$

For brevity, suppose a_e_, b_e_, and T_e_ are as shown in Equation (21):

(21) $$a_{e}=\left[\begin{array}{c} X\\ Y\\ Z \end{array}\right],b_{e}=\left[\begin{array}{c} x\\ y\\ z \end{array}\right],T_{e}=\left[\begin{array}{c} x_{0}\\ y_{0}\\ z_{0} \end{array}\right],$$

Then Equation (20) can be simplified to Equation (22):

(22) $$E=a_{e}-R\cdot b_{e}-T_{e},$$

Derived from the matrix of least squares, the error matrix E can be minimized under the conditions of (23), where ||.||_2_ represents the second-order norm:

(23) $$\min{\left({\left\|a_{e}-R\cdot b_{e}-T_{e}\right\|}_{2}\right)}^{2},$$

Equation (23) is unfolded, as shown in Equation (24):

(24) $${\left({\left\|a_{e}-R\cdot b_{e}-T_{e}\right\|}_{2}\right)}^{2}={\left(a_{e}-R\cdot b_{e}-T_{e}\right)}^{T}\cdot (a_{e}-R\cdot b_{e}-T_{e}),$$

The partial derivative of Equation (24) with respect to the translation vector T_e_ is obtained and makes its partial derivative equal to zero. When the error matrix E reaches the minimum, the optimal solution of the translation vector T_e_ is shown in Equation (25):

(25) $$T_{e}=\frac{1}{n}\sum_{i=1}^{n}a_{e}-R\frac{1}{n}\sum_{i=1}^{n}b_{e},$$

According to Equation (25), we can calculate the center of gravity of multiple common points in two coordinate systems, respectively. Then, the optimal translation vector T_e_ can be calculated by using rotation matrix R and the gravity of multiple common points. 

## 4. Performance Evaluation

**Figure 5:**
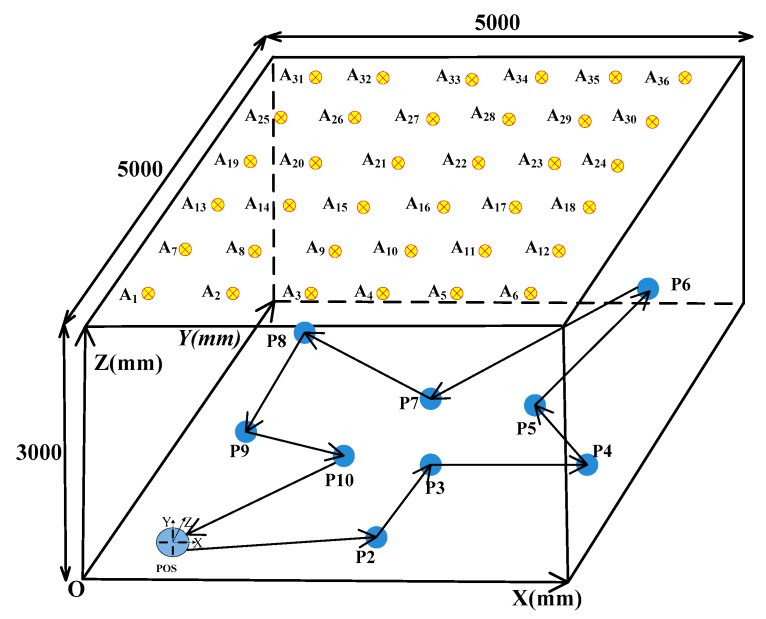
System model.

We evaluated the proposed method by using simulation. The simulation scenario is performed in an indoor system mode, and Figure 5 illustrates the scene of an indoor positioning and orientation measurement system.

As shown in Figure 5, the dimension of the system model is 5000 mm × 5000 mm × 3000 mm. Thirty-six infrared LEDs are fixed on the ceiling as anchors. Meanwhile, their coordinates in ICS are shown in Table 2. 

The POS moves under these anchors from P1 to P10 position, as shown in Figure 5. We set the P1 at [500 mm, 500 mm, 1000 mm] in ICS, and the x, y, z-axis rotation angle of POS in ICS are α = 0°, β = 0°, γ = −25°, so the translation vector T = [500 mm, 500 mm, 1000 mm]^T^. According to the coordinate of intersection points on linear CCD1, CCD2, CCD3, and CCD4, the anchors of A_1_, A_2_, and A_7_ are located in the FOV of the POS. 

According to the parameters of POS in Table 1, we can get the coordinate of intersection points of A_1_ on CCD1 is m_1_ = [78.79 mm, 0 mm, 0 mm]^T^ in POSCS, A_1_ on CCD2 is m_2_ = [0 mm, 77.35 mm, 0 mm]^T^ in POSCS, A_1_ on CCD3 is m_3_ = [−74.27 mm, 0 mm, 0 mm]^T^ in POSCS, A_1_ on CCD4 is m_4_ = [0 mm, −75.70 mm, 0 mm]^T^ in POSCS. Similarly, the coordinate of intersection points of A_2_ and A_7_ on CCD1, CCD2, CCD3, CCD4 are n_1_, n_2_, n_3_, n_4_ and p_1_, p_2_, p_3_, p_4_ in POSCS, as shown in Table 3. 

If we compare the coordinate values of these calculated projection point m_1_, m_2_, m_3_, m_4_, n_1_, n_2_, n_3_, n_4_, p_1_, p_2_, p_3_, p_4_ with the simulation parameters in Table 1, only p_4_ is not within the measurable range of the CCD4 in POSCS. However, three projection points on four linear CCDs are enough to reconstruct the 3D coordinates of this anchor in POSCS, and the additional projection point can be used as redundancy. Although p_4_ is not located in the measurable range of the CCD4 in POSCS, it does not affect the POS to estimate the coordinate of A_7_ in POSCS. This means that even if one of the ODIUs malfunctions, it will not affect the normal operation of the entire system. Thus, we will use m_1_, m_2_, m_3_, n_1_, n_2_, n_3_, p_1_, p_2_, p_3_ to estimate the coordinate of A_1_, A_2_, and A_7_ in POSCS. 

Based on the coordinate of m_1_, m_2_, and m_3_, we can estimate the coordinate of A_1_ in POSCS is a_1_= [−110.75 mm, −40.31 mm, 2499.99 mm] ^T^. Similarly, according to n_1_, n_2_, n_3_, and p_1_, p_2_, p_3_ we can estimate the coordinate of A_2_ and A_7_ in POSCS are a_2_= [644.50 mm, −392.49 mm, 2499.99 mm]^T^ and a_3_= [241.43 mm, 714.95 mm, 2499.99 mm]^T^, as shown in Table 4. 

According to the 3D coordinates of the three anchors A_1_, A_2_, A_7_ in ICS and POSCS, as shown in Table 2 and Table 4, we can use the Rodrigues coordinate transformation algorithm in Section 3 to estimate the position and orientation of the POS at the P1 position. 

Similarly, according to the set value from P2 to P10 position, following the steps at the P1 position, we can acquire all measurable anchors, and the results are shown in Table 5.

According to Table 5, we can estimate the coordinate of the measurable anchors in POSCS, and the position and orientation from P1 to P10 are listed in Table 6. 

The maximum measurement error and average measurement error of x, y, and z-axis are calculated based on Table 6, and then the average error is used to calculate the standard deviation, as shown in Table 7. 

Similarly, the maximum measurement error, average measurement error, and standard deviation of pitch, roll, and yaw angle are shown in Table 8. 

According to the evaluation results of measurement error in Table 7 and Table 8, the method proposed in this paper is compared with [15], as shown in Table 9. The measurement error in our method is smaller than the off-the-shelf methods, which indicates the feasibility and high accuracy of the indoor position and orientation measurement method proposed in this paper. 

## 5. Discussion

Indoor navigation is critical for developing intelligent carriers. We design a novel POS by using four pairs of linear CCDs and cylindrical lenses for indoor navigation without the assistance of an IMU or other costly devices. This design has the following advantages: (1) The POS is designed for the indoor position and attitude measurement purposes in a wide range, and achieves sufficient accuracy in the structured indoor environment. (2) Our method does not need to consider the 2D or 3D working situation, respectively. We can estimate the position and orientation in a 3D situation or the position and direction in a 2D situation, as long as a minimum of three anchors is within the field of vision of the designed POS. Meanwhile, after three LED anchors are detected, we can estimate the position and orientation simultaneously, while many indoor positioning methods can only estimate the position. (3) Once the LED’s control unit and POS’s control unit are simultaneously triggered, and they will have an identical time sequence. Therefore, the number of POS used indoors is not limited, theoretically. (4) The structure of the proposed POS is a redundant system, even if a linear CCD is broken down; it does not affect the normal operation of the entire system, as long as the remaining three linear CCDs could work normally. 

Of course, there is still some idealization in the simulation design process. In order to demonstrate the feasibility and effectiveness of the whole system, we ignore the partial distortion and nonlinear effects of the cylindrical lens. However, it depends entirely on the perfection of the cylindrical lens design and the cost, which is beyond the scope of this paper [29]. 

## 6. Conclusions

In this paper, a high-precision three-dimensional indoor position and orientation measurement method is proposed, which can be used for indoor navigation. We design a new-style indoor position orientation sensor by using four pairs of linear CCDs and cylindrical lenses, and analyze the field of vision of the position orientation sensor. The proposed indoor POS can estimate the 3D coordinate of the infrared LED anchors, after acquiring the coordinate value of at least three anchors sequentially in the POSCS, the Rodrigues coordinate transformation algorithm is used to estimate the position and orientation of the POS simultaneously. The position and orientation measurement method is performed via simulation. Thirty-six infrared LEDs are employed in simulation model space with dimensions of 5000 mm × 5000 mm × 3000 mm. The maximum value of position error and orientation error during simulation are 0.06 mm and 0.01°, respectively. The simulation result indicates the feasibility and high accuracy of the proposed method. The POS proposed in this paper is robust and can resist certain system failures, which is an alternative for indoor navigation. 

There are two fundamental steps for the whole system to work properly. In this paper, we mainly elaborate on the POS operating principle and demonstrate the feasibility and high accuracy of the designed POS. In order to achieve a wide-range measurement of the indoor positioning and orientation, many anchor nodes will be fixed on the ceiling. However, the designed POS can only measure one anchor at a time in its FOV, so in the second phase, we will study how to design the wireless high-speed synchronous exposure between the infrared LED array and the POS. Meanwhile, we will conduct experiments to verify the proposed methods. Additionally, the architecture of the proposed POS is symmetrical and redundant, and we will study how to realize indoor position and orientation measurements without synchronization. 

Due to the fast scanning, high-speed data processing, and precise coordinate measurement characteristics of the linear CCD, this system will be the appropriate candidate for the indoor position and orientation estimation, especially for the fasting moving object such as the flying agent indoors.

## Figures and Tables

**Table 1:** Simulation Parameters.

Parameter	Specification (mm)	Parameter	Specification (mm)
CCD1_In	[60, 0, 0]	a1	[75, 30, 50]
CCD1_Out	[90, 0, 0]	b1	[75, −30, 50]
CCD2_In	[0, 60, 0]	a2	[−30, 75, 50]
CCD2_Out	[0, 90, 0]	b2	[30, 75, 50]
CCD3_In	[−60, 0, 0]	a3	[−75, −30, 50]
CCD3_Out	[−90, 0, 0]	b3	[−75, 30, 50]
CCD4_In	[0, −60, 0]	a4	[30, −75, 50]
CCD4_Out	[0, −90, 0]	b4	[−30, −75, 50]
focal length	50	d	60
sensing length	30		

**Table 2:** The coordinates of anchors in ICS.

Anchor	Coordinate (mm)	Anchor	Coordinate (mm)
A_1_	[416.66, 416.66, 3000.00]	A_19_	[416.66, 2916.66, 3000.00]
A_2_	[1250.00, 416.66, 3000.00]	A_20_	[1250.00, 2916.66, 3000.00]
A_3_	[2083.33, 416.66, 3000.00]	A_21_	[2083.33, 2916.66, 3000.00]
A_4_	[2916.66, 416.66, 3000.00]	A_22_	[2916.66, 2916.66, 3000.00]
A_5_	[3750.00, 416.66, 3000.00]	A_23_	[3750.00, 2916.66, 3000.00]
A_6_	[4583.33, 416.66, 3000.00]	A_24_	[4583.33, 2916.66, 3000.00]
A_7_	[416.66, 1250.00, 3000.00]	A_25_	[416.66, 3750.00, 3000.00]
A_8_	[1250.00, 1250.00, 3000.00]	A_26_	[1250.00, 3750.00, 3000.00]
A_9_	[2083.33, 1250.00, 3000.00]	A_27_	[2083.33, 3750.00, 3000.00]
A_10_	[2916.66, 1250.00, 3000.00]	A_28_	[2916.66, 3750.00, 3000.00]
A_11_	[3750.00, 1250.00, 3000.00]	A_29_	[3750.00, 3750.00, 3000.00]
A_12_	[4583.33, 1250.00,3000.00]	A_30_	[4583.33, 3750.00, 3000.00]
A_13_	[416.66, 2083.33, 3000.00]	A_31_	[416.66, 4583.33, 3000.00]
A_14_	[1250.00, 2083.33, 3000.00]	A_32_	[1250.00, 4583.33, 3000.00]
A_15_	[2083.33, 2083.33, 3000.00]	A_33_	[2083.33, 4583.33, 3000.00]
A_16_	[2916.66, 2083.33, 3000.00]	A_34_	[2916.66, 4583.33, 3000.00]
A_17_	[3750.00, 2083.33, 3000.00]	A_35_	[3750.00, 4583.33, 3000.00]
A_18_	[4583.33, 2083.33, 3000.00]	A_36_	[4583.33, 4583.33, 3000.00]

**Table 3:** The intersection point on CCD1-CCD4 in POSCS.

Intersection	Coordinate (mm)	Intersection	Coordinate (mm)
m_1_	[78.79, 0.00, 0.00]	m_2_	[0.00, 77.35, 0.00]
m_3_	[−74.27, 0.00, 0.00]	m_4_	[0.00, −75.70, 0.00]
n_1_	[63.37, 0.00, 0.00]	n_2_	[0.00, 84.54, 0.00]
n_3_	[−89.68, 0.00, 0.00]	n_4_	[0.00, −68.52, 0.00]
p_1_	[71.60, 0.00, 0.00]	p_2_	[0.00, 61.93, 0.00]
p_3_	[−81.45, 0.00, 0.00]	p_4_	[0.00, −91.12, 0.00]

**Table 4:** The coordinate of anchors in POSCS.

Anchor	Coordinate (mm)
a_1_ (A_1_)	−110.75, −40.31, 2499.99
a_2_ (A_2_)	644.50, −392.49, 2499.99
a_7_ (A_7_)	241.43, 714.95, 2499.99

**Table 5:** Measurable anchors from P1 to P10.

Position	Anchors in FOV
P1	A_1_, A_2_, A_7_
P2	A_15_, A_16_, A_21_, A_22_, A_23_, A_28_
P3	A_20_, A_21_, A_26_
P4	A_24_, A_29_, A_30_
P5	A_17_, A_18_, A_23_, A_24_
P6	A_16_, A_22_, A_23_, A_29_
P7	A_1_, A_7_, A_8_, A_13_, A_14_, A_15_, A_20_
P8	A_15_, A_20_, A_21_, A_26_, A_27_
P9	A_25_, A_26_, A_31_, A_32_
P10	A_15_, A_16_, A_21_, A_22_, A_23_, A_27_, A_28_

**Table 6:** The position and orientation of POS.

	Position (mm)	Orientation(°)		
		Pitch(α)	Roll(β)	Yaw(γ)
P1 Set value	[500.00, 500.00, 500.00]	0.00	0.00	−25.00
P1 estimated value	[500.00, 499.99, 499.98]	0.00	0.00	−24.99
P2 Set value	[2450.00, 1180.00, 610.00]	30.00	−5.00	−45.00
P2 estimated value	[2449.99, 1179.99, 609.98]	29.99	−5.00	−45.00
P3 Set value	[2917.00, 2345.00, 1234.00]	16.00	27.00	90.00
P3 estimated value	[2917.00, 2344.98, 1233.99]	16.00	26.99	89.99
P4 Set value	[4763.00, 2406.00, 1816.00]	37.00	16.00	−125.00
P4 estimated value	[4762.99, 2405.99, 1816.00]	37.00	15.99	−125.00
P5 Set value	[4686.00, 2758.00, 1201.00]	−8.00	15.00	−33.00
P5 estimated value	[4686.01, 2758.00, 1200.98]	−8.01	15.00	−33.00
P6 Set value	[4700.00, 4716.00, 1768.00]	−35.00	35.00	0.00
P6 estimated value	[4699.97, 4716.00, 1767.99]	−35.00	34.99	0.00
P7 Set value	[2615.00, 2934.00, 436.00]	−18.00	30.00	−15.00
P7 estimated value	[2615.00, 2934.01, 435.99]	−18.00	30.00	−14.99
P8 Set value	[700.00, 4612.00, 1300.00]	−33.00	−28.00	0.00
P8 estimated value	[699.98, 4612.00, 1299.99]	−33.00	−28.00	0.00
P9 Set value	[600.00, 2500.00, 968.00]	40.00	0.00	90.00
P9 estimated value	[599.98, 2499.94, 967.99]	40.00	0.00	89.99
P10 Set value	[1500.00, 1300.00, 712.00	28.00	−23.00	42.00
P10 estimated value	[1499.99, 1299.97, 712.00]	27.99	−23.00	42.00

**Table 7:** Evaluation of Position Measurement.

	Maximum Error (mm)	Average Error (mm)	Standard Deviation (mm)
x	0.030	0.011	0.0099
y	0.060	0.015	0.0184
z	0.020	0.011	0.0074

**Table 8:** Evaluation of Orientation measurement.

	Maximum Error (°)	Average Error (°)	Standard Deviation (°)
Pitch (α)	0.010	0.003	0.0048
Roll (β)	0.010	0.003	0.0048
Yaw (γ)	0.010	0.004	0.0052

**Table 9:** Error comparison between two measurement methods.

	Position Maximum Error (mm)	Orientation Maximum Error (°)
Paper [15] method	3.80	0.104
Our method	0.06	0.010

## References

[1] Heibmeyer S., Overmetyer L., Muller A. Indoor positioning of vehicles using an active optical infrastructure. Proceedings of the 2012 International Conference on Indoor Positioning and Indoor Navigation (IPIN).

[2] Lee H., Tak J., Choi J. (2017). Wearable antenna integrated into military berets for indoor/outdoor positioning system. IEEE Antennas Wirel. Propag. Lett..

[3] Perez L., Rodriguez I., Rodriguez N., Usamentiaga R., Garcia D.F. (2016). Robot guidance using machine vision techniques in industrial environments: A comparative review. Sensors.

[4] Ashraf I., Hur S., Park Y. (2019). Indoor positioning on disparate commercial smartphones using Wi-Fi access points coverage area. Sensors.

[5] Fan Q., Sun B., Sun Y., Wu Y., Zhuang X. (2017). Data fusion for indoor mobile robot positioning based on tightly coupled INS/UWB. J. Navig..

[6] Kuang J., Niu X., Chen X. (2018). Robust pedestrian dead reckoning based on MEMS-IMU for smartphones. Sensors.

[7] Deng Z., Yu Y., Yuan X., Wan N., Yang L. (2013). Situation and development tendency of indoor positioning. China Commun..

[8] Zafari F., Gkelias A., Leung K.K. (2019). A survey of indoor localization systems and technologies. IEEE Commun. Surv. Tutor..

[9] He S., Chan S.G. (2016). Wi-Fi fingerprint-based indoor positioning: Recent advances and comparisons. IEEE Commun. Surv. Tutor..

[10] Mazhar F., Khan M.G., Sallberg B. (2017). Precise indoor positioning using UWB: A review of methods, algorithms and implementations. Wirel. Pers. Commun..

[11] Qi J., Liu G.P. (2017). A robust high-accuracy ultrasound indoor positioning system based on a wireless sensor network. Sensors.

[12] Faragher R., Harle R. (2015). Location fingerprinting with bluetooth low energy beacons. IEEE J. Sel. Areas Commun..

[13] Kiraci E., Franciosa P., Turley G.A., Olifent A., Attridge A., Williams M.A. (2017). Moving towards in-line metrology: evaluation of a Laser Radar system for in-line dimensional inspection for automotive assembly systems. Int J. Adv. Manuf. Tech..

[14] Soe N. (2004). Feature Based Design for Jigless Assembly. Ph.D. Thesis.

[15] Huang Z., Zhu J., Yang L., Xue B., Wu J., Zhao Z. (2015). Accurate 3-D position and orientation method for indoor Mobile robot navigation based on photoelectric scanning. IEEE Trans. Instrum. Meas..

[16] Hijikata S., Terabayashi K., Umeda K. A simple indoor Self-Localization system using infrared LEDs. Proceedings of the 2009 Sixth International Conference on Networked Sensing Systems (INSS).

[17] Tian R., Li Q. Research on the application of rectangle object constraint in active vision of Mobile robot. Proceedings of the 2016 International Conference on Robotics and Automation Engineering (ICRAE).

[18] Gu D., Chen K.S. (2015). Design and performance evaluation of wiimote-based two-dimensional indoor localization systems for indoor mobile robot control. Measurement.

[19] Kohoutek T.K., Mautz R., Wegner J.D. (2013). Fusion of building information and range imaging for autonomous location estimation in indoor environments. Sensors.

[20] Paredes J., Alvare F., Aguilera T., Villadangos J. (2018). 3D indoor positioning of UAVs with spread spectrum ultrasound and time-of-flight cameras. Sensors.

[21] Merriaux P., Dupuis Y., Boutteau R., Vasseur P., Savatier X. (2017). A study of vicon system positioning performance. Sensors.

[22] Ai L., Yuan F., Ding Z. (2008). Measurement of spatial object’s exterior attitude based on linear CCD. Chin. Opt. Lett..

[23] Kumar A., Bentzvi P. (2016). Spatial object tracking system based on linear optical sensor arrays. IEEE Sens. J..

[24] Zhang Y., Liu C., Fu L., Liu H. A design of cylindrical lens for linear CCD used in dynamic envelope curve measurement of high-speed train. Proceedings of the 2015 International Conference on Optical Instruments and Technology.

[25] Wu J., Ding H., Wang G. Aberration analysis and adjustment of non-spherical lens in the linear CCDs three-dimensional measurement system. Proceedings of the Optical Fabrication, Testing, and Metrology.

[26] Wu J., Wen Q. The method of realizing the three-dimension positioning based on linear CCD sensor in general DSP chip. Proceedings of the 2008 30th Annual Internal Conference of the IEEE Engineering in Medicine and Biology Society.

[27] Yang F., Dai H., Xing H. Least squares based on Rodrigues Matrix and its application in similar material model of mining. Proceedings of the 2015 IEEE International Conference on Mechatronics and Automation (ICMA).

[28] Felus Y.A., Burtch R.C. (2009). On symmetrical three-dimensional datum conversion. GPS Solut..

[29] Liu H., Yang L., Guo Y., Guan R., Zhu J. (2015). Precise calibration of linear camera equipped with cylindrical lenses using a radial basis function-based mapping technique. Opt. Express.

